# Pediatric febrile urinary tract infections: the current state of play

**DOI:** 10.1186/1824-7288-37-57

**Published:** 2011-11-30

**Authors:** Ian K Hewitt, Giovanni Montini

**Affiliations:** 1 Department of Pediatrics, Princess Margaret Hospital for Children, Perth, Australia; 2 Department of Pediatrics, Azienda Ospedaliero-Universitaria Sant'Orsola-Malpighi Bologna, Italy

## Abstract

Studies undertaken in recent years have improved our understanding regarding the consequences and management of febrile urinary tract infections (UTIs), which are amongst the most common serious bacterial infections in childhood, with renal scarring a frequent outcome.

In the past pyelonephritic scarring of the kidney, often associated with vesico-ureteral reflux (reflux nephropathy) was considered a frequent cause of chronic renal insufficiency in children. Increasing recognition as a consequence of improved antenatal ultrasound, that the majority of these children had congenital renal hypo-dysplasia, has resulted in a number of studies examining treatment strategies and outcomes following UTI.

In recent years there is a developing consensus regarding the need for a less aggressive therapeutic approach with oral as opposed to intravenous antibiotics, and less invasive investigations, cystourethrography in particular, following an uncomplicated first febrile UTI. There does remain a concern that with this newer approach we may be missing a small subgroup of children more prone to develop severe kidney damage as a consequence of pyelonephritis, and in whom some form of intervention may prove beneficial. These concerns have meant that development of a universally accepted diagnostic protocol remains elusive.

## 

Febrile urinary tract infections (UTI) have long been considered amongst the most common serious bacterial infections in childhood with renal scarring a frequent outcome [1]. We have recently published a review on this topic, highlighting developments in our understanding as a consequence of research conducted in this field, particularly during the past decade [2].

The recognition of an association between UTI, the presence of vesicoureteral reflux (VUR), and the propensity to develop pyelonephritis with subsequent renal scarring led to the concept of "reflux nephropathy" [3]. This term implied a causal relationship between VUR, kidney infection and subsequent major renal damage. As a consequence aggressive treatment with intravenous antibiotics, followed by extensive investigation, initially with urography, later with ultrasound (US) and radionucleotide scintigraphy to detect renal scarring, as well as voiding cystourethrography (VCUG) to detect and grade VUR, was strongly encouraged [4]. Furthermore, children with VUR were advised to commence long term antibiotic prophylaxis or undergo surgical correction of the reflux [5].

More recently, assumptions inherent in the above approach to UTIs have been questioned. Large registries, documenting progression to end-stage kidney disease, failed to show any reduction in the incidence or frequency as a consequence of aggressive investigation and management of UTIs [6]. Improved antenatal US has led to the recognition that congenital abnormalities of the kidneys and urinary tract (CAKUT), hypo-dysplasia with or without VUR in particular, is more significant than acquired pyelonephritic scarring as a causative factor for chronic renal insufficiency [7].

In recent years, a number of important prospective clinical trials have improved our understanding, and led us to critically evaluate previous clinical interventions and subsequent investigative protocols.

Studies conducted in infants and young children with uncomplicated febrile UTIs, demonstrated oral antibiotics were equally effective as intravenous antibiotics for the treatment of pyelonephritis [8,9]. There was no significant difference in the time to defervescence or rates of subsequent renal scarring. This allows the potential for considerable cost savings.

Antibiotic prophylaxis, widely administered to prevent recurrent UTIs, particularly in the presence of reflux, was the next to undergo critical appraisal. Four prospective studies involving 899 children with absent or lesser grades of VUR demonstrated similar rates of recurrent UTI in the treatment versus no treatment groups [10-13], with no significant difference in the incidence of late scarring in 2 of the studies [10,13]. The first large scale randomised, placebo controlled trial involving 576 children with absent or any grade of reflux demonstrated a marginal benefit of antibiotic prophylaxis, however required 14 patient years of treatment to prevent one UTI [14]. Recently the Swedish Reflux Study [15] reported on 203 children aged 1 to 2 years with grade III-IV dilating VUR, who were randomised to antibiotic prophylaxis, surgical correction or observation. A reduced rate of recurrent UTI was found in girls in the treatment groups compared with observation. In addition no scars occurred in girls on prophylaxis, while those following surgical intervention or in observation suffered 13 scars. No treatment benefit was observed in boys.

In these and other prospective studies of febrile UTI with previously healthy kidneys, around 60% develop pyelonephritis, of whom 10-40% will have subsequent scarring [2]. The scarring is usually minor, rarely causing a reduction in overall kidney function, with the severity appearing unrelated to the age of the child [16,17].

These and other studies have led to a general consensus regarding the need to temper the investigations following an uncomplicated first febrile UTI. In the past 5 years a range of approaches have been promoted [18-21]. While no agreement as to an optimal diagnostic protocol exists, there has been a shift away from invasive procedures and those with a substantial radiation burden. In particular, the need to detect all but the few cases of high grade dilating VUR has been questioned, leading to a marked reduction in the number of VCUGs performed. Ultrasound remains widely recommended as a safe non-invasive procedure, however the widespread use of antenatal US has meant that this modality rarely detects abnormalities that result in the need for intervention, and appears of limited value following a first febrile UTI [22,23]. With an increasing focus on the single adverse long term outcome of renal scarring, radionucleotide scintigraphy employing technetium^99 ^dimercaptosuccinic acid (DMSA) scanning has been recommended by some. The scan is conducted either in the acute phase following a UTI to confirm pyelonephritis in which case further follow-up and investigation is advised, the "Top Down" approach [18]; or 6 to 12 months later to confirm the presence of any scarring that might require ongoing review [23].

There remain a number of significant unresolved issues.

The development of a universally accepted diagnostic protocol remains elusive. Such a protocol should detect the very few children at risk of progressive kidney damage, who have the propensity to develop the complications of hypertension, pre-eclampsia and chronic renal insufficiency; at the same time not subjecting the vast majority of otherwise healthy children to invasive, costly and unnecessary interventions.

Many remain concerned that we may be missing a small subgroup of children more prone to develop severe kidney damage as a consequence of pyelonephritis, and in whom some form of intervention may prove beneficial. The possibility that various forms of renal hypo-dysplasia, with and without cystic change, may predispose to severe scarring as a consequence of pyelonephritis has not been critically evaluated. The complex interaction between urinary tract obstruction, dysfunctional voiding, increased bladder pressure profiles and the infection-inflammation process characterising pyelonephritis has long been recognised as a significant issue in progression toward chronic renal insufficiency, with current intervention strategies often palliative at most.

Current research is being undertaken to better understand the genetic predisposition and embryologic mechanisms underlying CAKUT in addition to factors that might further complicate acquired pyelonephritic scarring, such as genes involved in inflammation and fibrosis as well as hypertension [24-27]. Embryologic studies suggest abnormal laterally displaced ureteral budding from the bladder wall may predispose to a dysplastic refluxing ureter that makes contact with a reduced amount of mesenchyme forming a smaller hypo-dysplastic poorly functioning kidney.

Finally future strategies to prevent or ameliorate the kidney damage caused by pyelonephritis need to be explored. A recent publication [28] examining steroid therapy in the acute phase of the infection suggests some benefit as previously reported in animal models [29].

The "never ending story" of febrile UTIs has received a lot of attention in recent times, resulting in change from an aggressive to a more circumspect but also watchful approach to the problem. Physicians should however remain alert to the possible diagnosis of pyelonephritis in febrile children and treat appropriately. Where there are atypical features such as lack of response to appropriate antibiotics, infection with non E. Coli organisms, or recurrent episodes further evaluation is always warranted.
